# 2.M. Workshop: Empowering examples of high vaccination in minorities: learning from three underserved communities

**DOI:** 10.1093/eurpub/ckac129.104

**Published:** 2022-10-25

**Authors:** Organised by: RIVER-EU Project, Chair persons: Michael Edelstein (Israel), Danielle Jansen (EUPHA-CAPH)

## Abstract

Childhood vaccine uptake in most minority or ethnic communities in Europe is typically substantially and unacceptably lower compared to the general population. Ethnic, religious, or cultural minorities are more likely to encounter health system barriers to accessing health care services which are a major contributor to comparatively low vaccine uptake among these disadvantaged communities. Despite these challenges, some minority communities manage to achieve high uptake for childhood immunization sometimes higher than the general population in their respective countries. We have called these populations “empowering examples”. As part of RIVER-EU (Reducing Inequalities in Vaccine uptake in the European region - Engaging Underserved communities) an EU funded project aiming to improve access to vaccine services for children and youth, reducing inequality and improving vaccine uptake of HPV and MMR vaccination in underserved communities in Europe, we collected evidence on health system barriers and enablers to vaccination among underserved communities, including three empowering examples of underserved minority communities that achieve high vaccine uptake: the Somali community in Finland, the Arab community in Israel, and the Bangladeshi community in the United Kingdom. Understanding enablers to vaccination in these populations will help generate knowledge that will be translated to other populations in order to facilitate similar enablers, thus improving vaccine uptake. The main objective of this workshop is to share and discuss the preliminary findings related to health system enablers from these three “empowering examples.” The workshop will be structured in 3 presentations of 12 minutes each. Each empowering example will present preliminary findings from interviews and focus group sessions conducted with each minority community. Presentations will be followed by a moderated questions and answers session that will help the audience understand what factors have contributed high vaccine uptake in these populations and how they can be applied to others. Participants will be invited to share experiences from their own countries.

**Key messages:**

• Accessibility, trust in the health care system, and perceived vaccine safety, were found to be powerful health system enablers to HPV and MMR vaccination among three underserved populations studied.

• Normalizing vaccination as “just something you do” seems to be a common approach among the highly vaccinated minorities.

